# BUGS in the Analysis of Biodiversity Experiments: Species Richness and Composition Are of Similar Importance for Grassland Productivity

**DOI:** 10.1371/journal.pone.0017434

**Published:** 2011-03-02

**Authors:** Andy Hector, Thomas Bell, Yann Hautier, Forest Isbell, Marc Kéry, Peter B. Reich, Jasper van Ruijven, Bernhard Schmid

**Affiliations:** 1 Institute of Evolutionary Biology and Environmental Studies, University of Zurich, Zurich, Switzerland; 2 Department of Zoology, University of Oxford, Oxford, United Kingdom; 3 Department of Biology, McGill University, Montreal, Quebec, Canada; 4 Department of Forest Resources, University of Minnesota, St. Paul, Minnesota, United States of America; 5 Nature Conservation and Plant Ecology group, Wageningen University and Research Centre, Wageningen, the Netherlands; 6 Center for Population Analysis, Swiss Ornithological Institute, Sempach, Switzerland; Dalhousie University, Canada

## Abstract

The idea that species diversity can influence ecosystem functioning has been controversial and its importance relative to compositional effects hotly debated. Unfortunately, assessing the relative importance of different explanatory variables in complex linear models is not simple. In this paper we assess the relative importance of species richness and species composition in a multilevel model analysis of net aboveground biomass production in grassland biodiversity experiments by estimating variance components for all explanatory variables. We compare the variance components using a recently introduced graphical Bayesian ANOVA. We show that while the use of test statistics and the R^2^ gives contradictory assessments, the variance components analysis reveals that species richness and composition are of roughly similar importance for primary productivity in grassland biodiversity experiments.

## Introduction

Concern over the ongoing loss of biodiversity has prompted ecologists to investigate the consequences for ecosystem processes [1], [2]. This biodiversity and ecosystem functioning research has been one of the most hotly discussed topics in ecology since the mid-1990s. Much of the research within this area has used biodiversity-functioning experiments (biodiversity experiments for short) that assemble communities of differing diversity and monitor the response of different ecosystem processes (other approaches include diversity removal experiments and observational comparative surveys). Many of these biodiversity experiments feature replicated manipulations of more than one aspect of biodiversity raising the issue of how best to compare their relative importance. One particular area of debate has been over the importance of species richness – numbers of species - relative to other aspects of biodiversity, particularly composition – types of species [3], [4], [5], [6]. The terminology in this area can be confusing; to be clear, here we use composition to mean all the different combinations (mixtures and monocultures) of species used in a biodiversity experiment (that is, in our analysis composition is a factor with a level for each different combination of species). We do not examine species identity effects, by which we mean the (binary) presence or absence of each species across a range of mixtures.

In this paper we extend existing analyses of data from grassland biodiversity experiments (Table 1) to derive new insights. We compare a typical least squares mixed model ANOVA (“mixed model ANOVA”) with a maximum likelihood mixed-effects model (“mixed-effects model”) that treats some variables as fixed and some as random [7], [8] and a hierarchical or multilevel model (“multilevel model”) that calculates variance components for all variables [9]. Because mixed-effects models treat some variables as fixed and some as random comparing the relative importance of variables from these two different classes can be difficult, as we explain below. In contrast, multilevel models that calculate variance components for all variables (presented here as a graphical ANOVA table) allow for easier assessment of their relative importance [9], [10]. Using this recently suggested approach, we analyse data on aboveground annual net primary production (ANPP) from grassland biodiversity experiments and show that species richness and species composition are of similar importance for this ecosystem process.

**Table 1:** Summary of the relevant design details for the subsets of compatible data analysed from the grassland biodiversity experiments that replicated both species richness and composition (ordered by increasing aboveground annual net biomass production, ANPP).

Experimental Site	ANPP	Diversity	Compositions	Blocks	Plots	Year
Wageningen	149.9	1,8	9	6	54	3
BIODEPTH Portugal	200.2	1,2,4,8,14	27	1	56	2
BioCON	227.0	1,4,16	21	3	56	3
BIODEPTH Greece	232.4	1,2,4,8,18	26	2	52	2
BIODEPTH Sweden	255.7	1,2,4,8,12	28	2	58	2
Jena	453.4	1,2,4,8,16	78	4	156	2
BIODEPTH Switzerland	500.8	1,2,4,8,32	32	2	64	2
BIODEPTH Sheffield	528.8	1,2,4,8,12	26	2	54	2
BIODEPTH Silwood	564.1	1,2,4,8,11	33	2	66	2
BioGEN	621.5	1,4	16	1	32	2
BIODEPTH Ireland	630.7	1,2,3,4,8,	33	2	70	2
BIODEPTH Bayreuth	681.6	1,2,4,8,16	30	2	60	2
Total			359 (308 crossed)	29	778	

The subsets of the species richness gradients used are given by ‘Diversity’ and the number of species compositions (monocultures or polycultures) in each experiment by ‘Compositions’. For comparability we used data from the earlier stages of each experiment (year 2 or 3). Numbers of compositions for each experiment ignore duplication of species mixtures with other experiments but the row titled Total gives the number of compositions ignoring duplicates and discounting duplicates (in parentheses). The version of the variable with 308 levels was used in all analyses, resulting in partially crossed random effects for experimental sites and species composition in the mixed-effects model.

Biodiversity experiments have typically been analyzed within the classical least squares linear model framework that includes regression and analysis of variance (ANOVA) as special cases [11], [12], [13], [14]. Table 2 presents a typical analysis of data on aboveground biomass production from some of the major grassland biodiversity experiments. In ANOVA tables of this type, larger F values, with their accompanying lower P values, are often taken as evidence for greater importance of one explanatory variable relative to another. As we describe in the results in greater detail, species richness has an F value of 68.3 and species composition an F value of 1.3, which could be taken to imply that, on average, species richness was more important than species composition. However, test statistics and P values are a poor measure of relative importance of different explanatory variables since predictors with higher F ratios (and lower P values) do not necessarily correspond to effects with higher estimated magnitudes. The F value (variance ratio) for each explanatory variable is calculated by dividing its mean square - the signal - by the appropriate error, or noise, term. In many complex ANOVA designs (e.g. split-plot, repeated measures), including those that manipulate both species richness and composition, there are multiple treatment (signal) and error (noise) terms (as implied by the naming of multilevel or hierarchical models) and different explanatory variables are compared to different error terms. Therefore, in multilevel models F and P values are not useful for comparing the importance of explanatory variables that are tested against different error terms [9], [10].

**Table 2:** A typical least squares mixed-model ANOVA table of net annual aboveground biomass production in grassland biodiversity experiments.

Explanatory variable	DF	Sum of Squares	Mean Squares	F ratio	P (≥ F)	Error	R^2^ (%)
Experiment (E)	11	23053686	2095790	83.7	1.46×10^−12^	B	34
Block (B)	17	425869	25051	1.6	0.057	P	1
Species Richness (R)	1	6413444	6413444	68.3	4.88×10^−15^	C	9
Species Composition (C)	294	27608931	93908	1.3	0.144	E.C	41
Experiment*Richness (E.R)	11	1601422	145584	2.1	0.049	E.C	2
Experiment*Composition (E.C)	39	2762484	70833	4.6	1.59×10^−15^	P	4
Residual (plots) error (P)	387	5986016	15468				9
Total	760	67851852					100

Species richness (continuous, log_2_ scale) is the only fixed effect. The R^2^ - the percentage of the total sum of squares explained by each row of the table - is a limited measure of relative importance because it does not account for the large differences in degrees of freedom (DF). F or P values do not indicate relative importance because different explanatory variables are tested against different error terms (column 7): (1) Experiment (random) is tested against block; (2) Block (random) against the overall (between plot) residual error; (3) Species richness (fixed) against species composition; (4) Experiment*Richness (random) against Experiment*Composition; (5) Species composition (random) against its interaction with experiment; (6) Experiment*Composition (random) against the overall (between plot) residual error (following Hector et al. 1999 and Spehn et al. 2005).

An alternative measure of the relative importance of different explanatory variables is the increase (or decrease) in the multiple R-square (the proportion of the total sums of squares explained by a given variable) of a model when an explanatory variable is added (or removed). However, this also has limitations. First, conventional measures of R^2^ may not be appropriate for mixed models [9]. However, even if we ignore this problem the R^2^ may not be a good indicator or relative importance of different explanatory variables. For example, as we will discuss later, in Table 2 species richness explains 9 percent of the total sums of squares while species composition explains 41 percent. Comparison of the R^2^ values could be taken to imply that species composition is more important than species richness and therefore leads to a conclusion opposite to that given by the F and P values. However, this fails to take into account the large disparity in the degrees of freedom (1 for species richness when it is treated as a continuous predictor versus 294 for species composition).

R^2^ also has limitations for comparing the importance of species richness at different experimental sites since, all else being equal, R^2^ is positively related to the range of continuous explanatory variables [9]. In other words, all else being equal, experiments with a wider range of species richness will have higher R^2^s.

Variance components are a better quantity for comparing the relative importance of different explanatory variables [10], [15], [16]. Each variance (mean square) in an ANOVA table is made up of variation from different sources - the variance components. There is one special case where the variance and the variance component are equal, and that is for the residual mean square at the bottom of the ANOVA table. As we move up the ANOVA table the other mean squares are made up of this residual variance component plus one or more additional (or added) variance components. We illustrate this with regard to this example below but first detail several problems that must be overcome in order to get variance components for all explanatory variables.

Within the mixed-effects model framework, variance components are calculated only for random effects and not for fixed effects. This is an issue of interpretation, not calculation [17], [18], [19]: the variance component for a random effect is the variation estimated for the entire population of possible levels of the random effect (the so-called super population) whereas a variance component calculated for a fixed effect would reflect only the variation in the sample of levels used in that particular analysis (the so-called finite population) which may not extend to alternative versions of the design that use other levels of the fixed factor. However, the calculation of variance components for fixed and random effects would be identical, despite this difference in interpretation [18], [19]. This lack of variance components for fixed effects in mixed-effects models obviously means that they cannot be used to compare the importance of fixed and random effects.

There are no watertight definitions of fixed and random effects [9], [20] but the basic idea can be illustrated by contrasting experimental treatments with blocking variables [21]. Fixed effects are easier to explain since least squares analysis mostly treats variables as fixed (indeed random effects were not distinguished until more than 20 years after the birth of ANOVA [22]) and so they are familiar to all analysts as classical treatment variables. In contrast, we can consider the levels of a random effect to be a sample representative of the entire (super) population – of blocks for example. In mixed-effects models the individual values of the levels of a random factor are typically of less interest than quantifying the overall level of variation, although their individual values - the so-called Best Linear Unbiased Predictions (BLUPs) - can be examined. Blocking terms are a common example of random effects where we might be more interested in the estimated magnitude of the block-to-block variability than in the predictions for particular blocks.

Within the least squares framework, the variance components can be calculated from the means squares in an ANOVA table but only unambiguously for balanced designs. This is a major limitation of the least squares framework for analyses of this type. For unbalanced designs statisticians recommend estimating variance components using maximum likelihood methods. To make matters worse, even for least squares analyses of balanced datasets there is disagreement over the appropriate error term for use in tests for interactions between fixed and random effects, what Nelder [18], [19] called the “great mixed-model muddle”. Even for simple two-factor designs, there are two main schools of thought on how to do the testing plus some additional alternative points of view [23] that we explain briefly in the methods. We mention this because one advantage of maximum likelihood mixed-effects models is that there appears to be greater agreement regarding statistical testing [24]. Maximum likelihood mixed-effects models also have several other advantages over the classical least squares approach regarding better handling of missing values, parameter estimation and prediction [9]. Confusingly, modern mixed-effects models can be fitted using either standard maximum likelihood or restricted (reduced/residual) maximum likelihood (REML), an extension developed specifically for analyses with both fixed and random effects [20], [25].

While modern mixed-effects models can estimate variance components for unbalanced datasets, we are still left with the limitation that they are only calculated for the random effects due to the problems of interpretation explained above. This is a particular problem for the analysis of biodiversity experiments since species composition is usually treated as a random effect and species richness a fixed effect. Species composition is usually best treated as a random effect because replicate species compositions are usually only a small sample of all the possible combinations. In contrast, the species richness treatment is always treated as a fixed effect because its levels are deliberately selected (fixed) and form a larger fraction of the total range of possibilities. Because mixed-effects software generally does not calculate variance components for fixed effects there is no estimate for species richness that can be compared with that for species composition.

The solution suggested by Gelman [10] based on earlier ideas [17], [18], [19], [26] is straight-forward: we can simply calculate variance components for all variables (and interactions). This is generally not done in mixed-effects models because of the problem of interpretation: the variance components are always estimates for the super population and while this is appropriate for random effects it is generally thought of as inappropriate for fixed effects. To avoid this, Gelman's approach instead calculates finite-population variance components in all cases. The finite- and super-population variance components should have similar point estimates but the super-population estimates will have greater uncertainty, especially for variance components with few degrees of freedom. In other words, the intervals for the super population will be wider than for the finite population. Variance components can be presented on the variance (squared) scale but, for easier comparison with the point estimates, are often presented instead as standard deviations (the square-roots of the variance components) [8], [9]. For easy visual comparison we present the variance components on the standard deviation scale (the square roots of the variance components) with intervals following Gelman's graphical analogue of the classical ANOVA table. We can use the intervals to visually assess whether a variance component of zero is consistent with the data (note that the method does not allow estimates of exactly zero so that the intervals can only approach zero but not actually contain it). A similar analysis could be attempted by specifying all variables as random within a mixed-effects model with the caveat that the variance components are then the super population estimates and interpretation becomes more complex as explained above [27]. Gelman [9], [10] instead uses WinBUGS [28], [29], [30], one of the family of BUGS software (Bayesian inference Using Gibbs Sampling). In the literature, multilevel models include mixed-effects models, but to distinguish the two in this paper we use mixed-effects to refer to analyses containing both fixed and random effects (implemented using the R lmer function) and multilevel models to refer to our application of Gelman's approach – implemented using WinBUGS - that calculates variance components for all variables).

**Figure 1:**
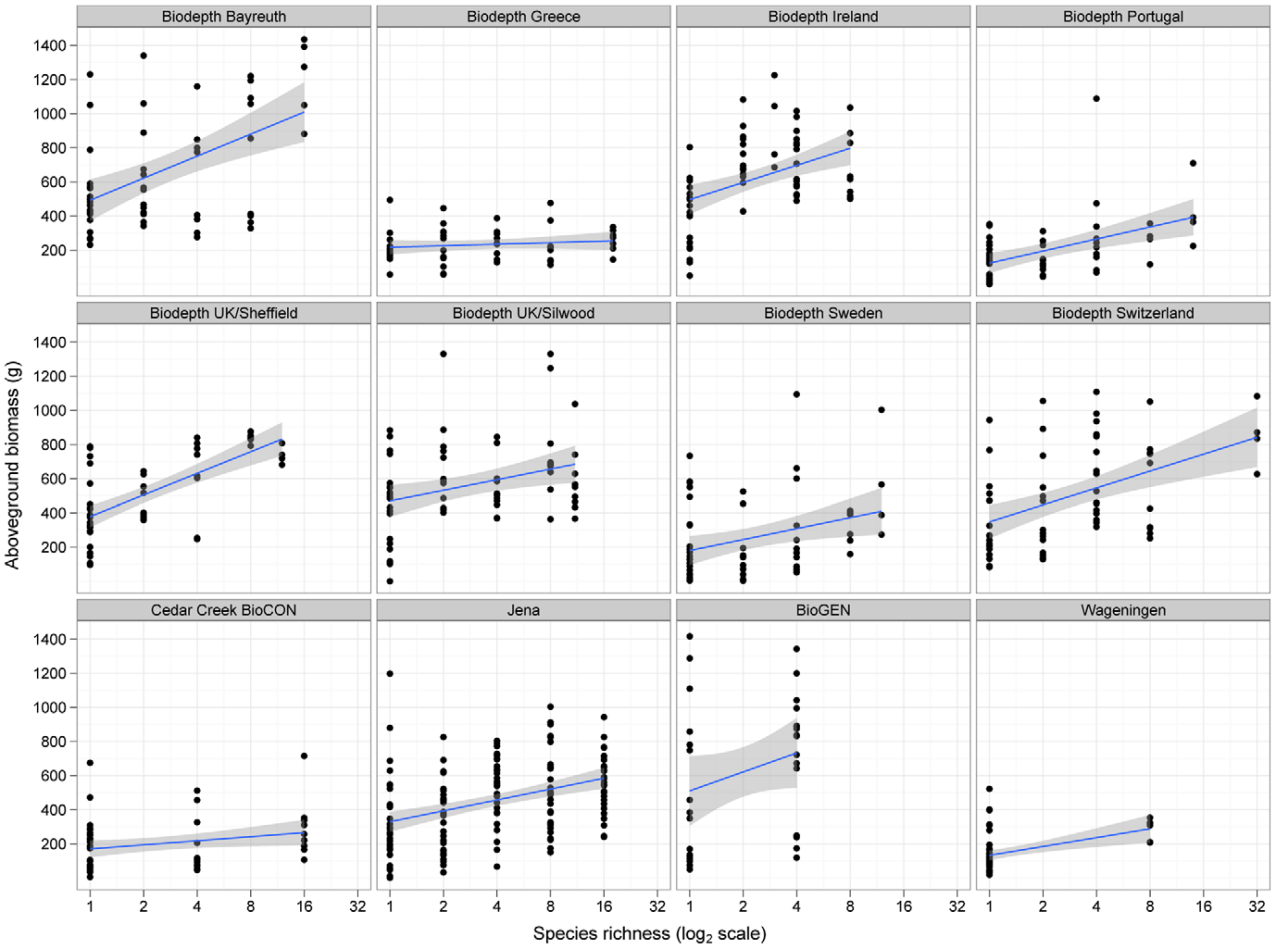
Regression slopes from the least squares mixed-model ANOVA. The response of aboveground annual net primary productivity to manipulations of species richness in each of the 12 grassland biodiversity experiments showing data for individual plots and fixed effects regression slopes fitted for each experiment with their 95% confidence intervals.

We applied the approach suggested by Gelman [9], [10] to data on aboveground annual net primary production from grassland biodiversity experiments that replicated both species richness and composition as part of their design (the eight BIODEPTH project sites, the Jena Biodiversity Experiment, the Wageningen Biodiversity Experiment, the BioGEN experiment and the BioCON experiment; Table 1). To perform the analysis we used WinBUGS linked to R 2.8.1 [31] via the R2winBUGS package [32] (Text S1). We analysed annual net primary production using a multilevel model containing explanatory variables for experimental sites (12 levels), blocks within experimental sites (29 levels), experimental communities with different species compositions (359 levels reduced to 308 after eliminating duplicate compositions at multiple sites), the diversity gradient at each experimental sites (11 points on a diversity gradient that is treated as a continuous replicated regression on the log_2_ scale) and the residual differences between plots (778 plots in total). Details of the experiments are given in Table 1 and the diversity gradients displayed in Figure 1.

In the following results section, we begin by first considering the results of the traditional ANOVA (Table 2), where the focus is on null hypothesis significance testing, before comparing and contrasting the results with the mixed-effects and multilevel model output that is focused more on estimation of the magnitude of the effects.

## Results

The least squares mixed-model ANOVA is summarized in Table 2 and the regression slopes from it are presented in Fig. 1 with their 95% confidence intervals and the observed aboveground ANPP of each plot. The slopes show a similar range of responses to previous comparative analyses in ranging from indistinguishable from zero to significantly positive with no negative slopes observed. Both the experiment-by-richness (variation in slopes) and the experiment-by-composition interactions are significant, suggesting that richness and composition are important but that the effects of both differ between experiments. Blocks have a much weaker effect that is not quite significant even with this large dataset. Closer examination of Table 2 confirms that because richness and composition are tested against different error terms their F and P values are of little use for comparing their relative importance. Similarly, Table 2 displays the big differences in degrees of freedom that (together with the multilevel nature of the analysis) also make the R^2^ values of limited use for comparing relative importance.

**Figure 2:**
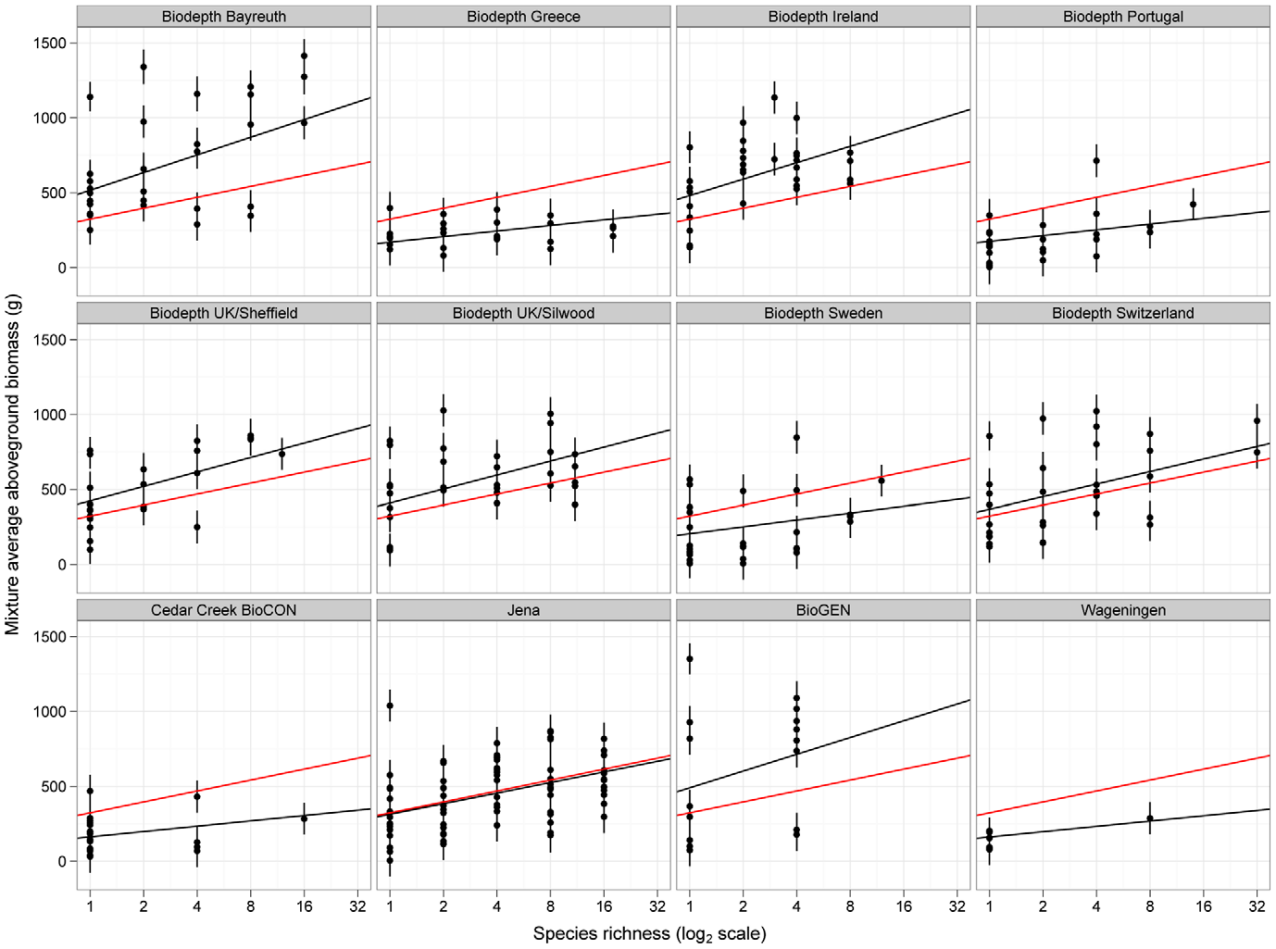
Regression slopes from the maximum likelihood mixed-effects model. The response of aboveground annual net primary productivity to manipulations of species richness in each of the 12 grassland biodiversity experiments showing average biomass for each species composition with their SEMs plus the overall average slope in red and the slope predicted for each site in black (with shrinkage) from the mixed-effects model.

Figure 2 illustrates some of the results from the mixed-effects model and some of the important differences from the least squares ANOVA. The regression slopes are the so-called Best Linear Unbiased Predictors (BLUPs) predicted by the mixed-effects model for each site [20]. The slopes are similar to those in Figure 1 but close inspection shows ‘shrinkage’ of the individual slopes towards the overall mean response (e.g. a slightly steeper slope in Greece). This occurs because the slope predicted for each site is a compromise (weighted average) of the value estimated for each site (as in Figure 1) and the overall average slope. Rather than display the individual plot values again as in Figure 1, Figure 2 displays the mean biomass predicted for each species composition together with their SEMs. Due to the separation of variables into fixed and random the summary of the analysis shown in Table 3 is split into two parts. The upper fixed-effects section reports the effects of (only) species richness in a manner similar to a classical ANOVA table (e.g. Table 2) except the R lmer function currently does not report a P value due to the difficulties in calculating them for mixed-effects models - although various methods are available for obtaining an approximate P value [33], [34]. The lower section for the random effects reports the variance components on the variance and standard deviation scales (the SDs are simply the square roots of the variance components) together with likelihood ratio tests of the change in deviance on removing each random effect in sequence from the full model. The estimates of the variance components can be used to compare the relative importance of the random effects but not for the fixed effect of species richness – hence the motivation for the multilevel model analysis that estimates variance components for all explanatory variables. For now, as well as noting the lack of a variance components for the fixed effects, also recall that the variance components are the super population estimates.

**Table 3:** Summary of the maximum likelihood mixed-effects model analysis reporting fixed- and random-effects separately.

Fixed effects	DF	SS	MS	F		
Species richness	1	441915	441915	28.6		

Random effects	Variance component	%	SD	logLikelihood	Chi^2^	P (≥ Chi^2^)
Experiment	21248.4	28	145.8	5087.5	260.1	<0.0001
Block	357.2	1	18.9	5217.8	1.9e^−5^	0.9966
Species composition	13951.9	18	118.1	5217.8	198.5	<0.0001
Experiment x Richness	1151.3	2	33.9	5118.5	20.6	<0.0001
Experiment x Composition	23751.5	31	154.115	5228.1	62.0	<0.0001
Residuals	15456.2	20	124.3			

The fixed effects are reported following the conventions of least squares ANOVA (e.g. Table 2). The R lmer function currently does not give P values due to the difficulties of calculating them for mixed-effects models. The random effects section reports the super-population variance components on the variance and standard deviation scales (the SD is simply the square root of the variance component) with likelihood ratio tests of the change in deviance on removing each random effect from the model in turn. Each variance component is expressed as a percentage of the summed total for all 6 six random effects (lower column 3: ‘%’).

**Figure 3:**
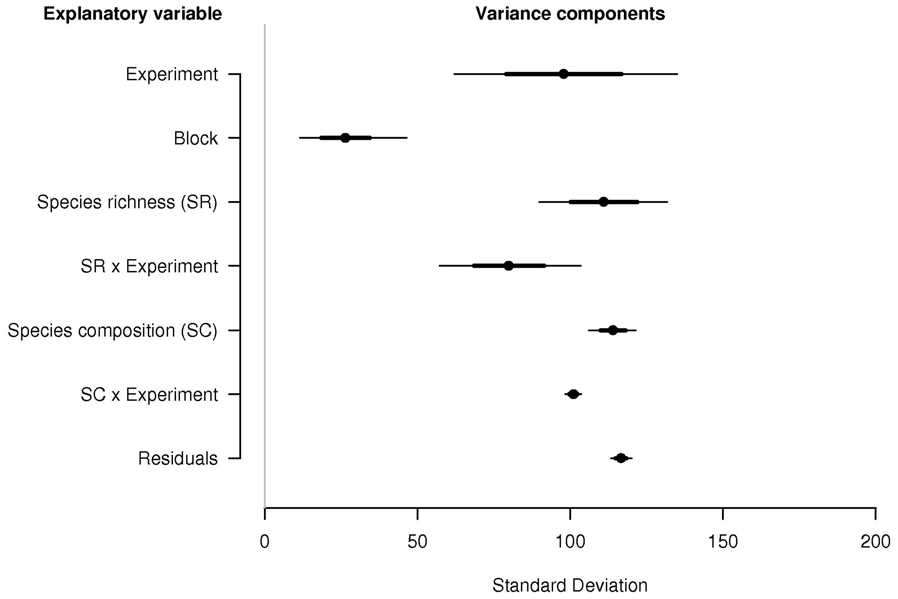
Variance components from the multilevel model analysis using BUGS presented as a graphical ANOVA table. Point estimates (standard deviation scale) are medians of the posterior distributions produced by Gibbs sampling using WinBUGS with 95% (wide) and 68% (narrow) intervals.

Finally, Figure 3 presents the results of multilevel model analysis. Since the motivation for the analysis is to compare the importance of species richness with species composition the point estimates of the variance components with 95 and 68% credible intervals (the Bayesian counterpart of confidence intervals [10]) are presented with more comprehensive output from the analysis given in Table 4. Before using the new method to compare the relative importance of species richness and composition it seems prudent to compare the results of the tests performed by the different methods. We have already seen that, despite some differences in the details, the least squares ANOVA and maximum likelihood mixed-effects model are qualitatively in agreement. Rows in the least squares ANOVA table (Table 2) and the fixed-effects summary of the mixed-effects model (Table 3, top) that contain non-significant F tests should correspond to estimates in the graphical ANOVA table (Figure 2) that are indistinguishable from zero. For our dataset, inferences based on the point estimates and intervals from the multilevel model (Figure 3) generally agree with those based on the least square ANOVA and mixed-effects model (Tables 2 and 3). The experiment-by-richness and the experiment-by-composition interactions are again significant (zero is well outside the confidence intervals) confirming that species richness and composition are important but that both vary among experiments. If, to compare the methods in greater detail, we progress to examining the main effects we can see that species richness is clearly significant in both approaches. The single exception is the main effect of the species composition that is judged significant in the graphical comparison of variance components and the likelihood ratio test of the random effect in the mixed-effects models but not by the F test in the conventional ANOVA table. However, in general tests of significance using the three different methods are in agreement, so we proceed to the main goal of comparing the relative importance of variables in the graphical Bayesian ANOVA table using the estimates of the variance components (on the standard deviation scale) from the multilevel model.

**Table 4:** Summary of the BUGS output for the multilevel model analysis.

Explanatory variable	Variance component(%) [SD scale]	SE	2.5%	16.0%	84.0%	97.5%
Experiment	96.5 (15)	19.1	59.4	77.1	115.3	136.0
Block	26.0 (4)	7.5	12.6	18.7	33.5	41.6
Species richness	111.2 (17)	11.3	88.2	100.3	121.4	133.5
Species composition	114.2 (18)	4.0	106.9	110.1	118.0	122.3
Experiment*Richness	80.3 (12)	11.6	57.3	68.6	92.1	103.0
Experiment*Composition	101.1 (16)	1.4	98.5	99.7	102.5	103.7
Residuals	116.5 (18)	1.7	113.2	114.8	118.3	119.8

Variance components are calculated as finite-population Standard Deviations and the percent contribution of each to the total are used as a rough measure of their relative importance. Column 3 gives the Standard Errors (SE) of the variance component estimates on the SD scale (given in column 2). The 2.5 and 97.5% quantiles are the upper and lower bounds of the 95% CI (Credible Interval) and the 16 and 84% values of a 68% CI (equivalent to ±1 SEM with symmetric normal distributions).

Using the estimation based approach we can immediately get a better sense of the relative importance of the different explanatory variables than is possible from a conventional ANOVA table or mixed-effects model. The variance components for species richness and composition and their interactions with experiment are all of a roughly similar magnitude, and of similar size to the unexplained variation. Only blocks stand out as relatively unimportant. The interaction of experiment with species richness is slightly smaller than the interaction of experiment with species composition suggesting the former is slightly more consistent (this effect was more pronounced in an earlier analysis before the final BioGEN dataset was added). In general, given the observed level of uncertainty, species richness and species composition (and experimental site too) are of similar importance for productivity in grassland biodiversity experiments.

## Discussion

The idea that losses of species richness could have negative impacts on ecosystem functioning initially met with a lot of resistance; in particular effects of richness were predicted to be minor compared to compositional effects [3], [4], [5], [35]. In this paper we present a new analysis of data on net aboveground biomass production in some of the major grassland biodiversity experiments that replicated both species richness and species composition, using a recently suggested application of multilevel model analysis to compare the explanatory variables in terms of their variance components. Counter to earlier predictions - and to unreliable impressions given by test statistics and the conventional R^2^ - the numbers and types of species present in experimental grassland communities are of roughly similar importance for aboveground productivity.

The analysis presented here compares the importance of species richness with that of species composition, that is the overall variation between the different species compositions used (i.e. the differences between all monocultures and polycultures). Our analysis is therefore limited to studies (sometimes subsets of the full data from each experiment) that replicated both species richness and composition. The comparison of species richness and composition is particularly problematical because species richness is a simple predictor with few degrees of freedom (1 when treated continuously) while species composition has many (294 here!) and so the variance components approach is particularly useful.

Many researchers interested in the effects of biodiversity on ecosystems focus on aspects of functional diversity including functional group richness (numbers of functional groups), functional composition (combinations of functional groups) and continuous measures of functional trait diversity [36], [37]. Comparing species richness with functional richness is less problematical when the degrees of freedom are the same and this has been done several times in the literature [38], [39], [40], [41], [42]. It was not possible to perform a combined analysis of functional diversity here as the 12 studies differ in this aspect of their designs and employ four different functional group classifications (for example, one of the eleven studies distinguishes species with the C3 or C4 photosynthetic systems, while another distinguishes short and tall herbs). Furthermore, the functional group effect would also be partially confounded with experiment since the Wageningen biodiversity experiment deliberately omitted all legume species to see whether biodiversity effects disappeared in their absence (they do not).

As explained above, one drawback of least squares mixed model ANOVA is its focus on null hypothesis significance testing and the associated difficulty in identifying the appropriate error terms, even for simpler examples than the relatively complex analysis presented here. One advantage of the Bayesian graphical ANOVA approach is that, by emphasizing estimation over testing, it sidesteps this ‘great mixed-model muddle’ – indeed, it does not require the analyst to specify error terms at all. Instead, inferences can be based upon the point estimates and intervals. In this analysis, the graphical comparison of variance components from the multilevel model (and the tests performed in the mixed-effects model analysis) support all of the conventional ANOVA tests except the one for the species composition main effect, questioning the assignment of the error term in this case. The point is moot in this case because the significant experiment-by-composition interaction term indicates the importance (albeit somewhat variable across experiments) of species composition but it illustrates how the automatic estimation-based testing possible with the graphical comparison of variance components might be a useful cross-check in the difficult assignment of error terms in the context of the ‘great mixed-model muddle’.

One point of discussion regarding statistical inference raised by the comparison between the mixed-effects and multilevel model is over the appropriate scale on which to compare the variance components. Mixed-effects analysis generally compares the variance components for the random effects as estimated on the variance scale, expressing each variance component as a percentage of the summed total [e.g.[16], [43]]. Some leading statisticians have even suggested that variance components calculated on the variance (squared) scale should be presented graphically as two dimensional areas rather than as a point estimates [26], in which case presenting variance components on the SD scale as point estimates with intervals seems appropriate. In contrast, the new graphical Bayesian ANOVA approach applied here compares the variance components as estimated on the standard deviation scale. Obviously, differences between the point estimates of variance components with larger and smaller effects will appear more pronounced on the variance scale than on the standard deviation scale (although obviously the upper and lower bounds of their intervals on the variance scale will be the square of those on the standard deviation scale too). So far as we can ascertain from the literature there seems to be no clear-cut argument in favour of one scale over the other but focusing on the standard deviation scale seems to be a recent trend even in the mixed-effects models literature [8]. It is also important to keep in mind that the variance components in the mixed-effects models are super-population estimates for the random effects only while the multilevel model analysis produces finite-population estimates for all explanatory variables.

Bayesian methods are increasingly recommended for use in statistical analysis in many areas of science including ecology [44], [45], [46], [47], [48]. However, they are both less widely understood than classical least squares methods and controversial [49], [50], [51]. The BUGS analysis presented here is Bayesian in that it uses Gibbs sampling to calculate posterior distributions. It is also Bayesian in treating all predictors as random variables described by a prior distribution. However, it is not Bayesian in the fullest sense of using informative prior values for the distributions of the random variables. Furthermore, while this approach is Bayesian in the ways described above, it is also more general in the sense that it is partly descended from similar ideas developed within the least squares and maximum likelihood frameworks [17], [18], [19], [26].

The use of variance components to assess the importance of different explanatory variables is not new, even in ecology [15], [52]. Indeed, even the graphical Bayesian ANOVA employed here has been used once before in ecology [53]. The new contribution of this paper is in using this methodology to reveal that, counter to earlier predictions, species richness and composition are of similar importance for primary production in grassland biodiversity experiments.

## Materials and Methods

### Experimental Design

We analyse compatible subsets of data from the grassland biodiversity field experiments that replicated species richness and composition as part of their designs. The conditions for inclusion were a species richness gradient with a minimum of two diversity levels that was composed of species composition (polycultures and monocultures) that were also replicated in a minimum of two plots. Since two of the 12 studies had only two levels of diversity where species composition was also replicated (Wageningen, 1 and 8 species; BioGEN, 1 and 4 species) we restricted the model to fitting simple linear regressions only (the linear regression with species richness on a log_2_ scale approach that has proved appropriate for the analysis of many biodiversity experiments). While the level of compositional replication is sometimes low with regard to estimating the effects of a particular species composition our focus here is on estimating the overall variability across all species compositions, that is, the variance component for species composition.

The eleven datasets combined for analysis here are the eight BIODEPTH experiments (Spehn et al. 2005), the Jena Biodiversity Experiment [54], [55], the Wageningen Biodiversity Experiment [56], [57], [58], [59], [60] the Cedar Creek BioCON experiment [42], [61], [62] and the BioGEN experiment [63]. The dataset is available online as supplementary material (Text S2). We analyze the response of aboveground ANPP (g m^−2^ year^−1^) of individual plots at each of the 12 experimental sites. Details of the experimental designs are given in Table 1 and the supporting citations.

### Analysis

We employed three related analyses: a least squares mixed-model ANOVA, a maximum likelihood mixed-effects analysis and a Bayesian multilevel (or hierarchical) model using Gibbs Sampling (MCMC). As the multilevel model is the newest and least familiar we focus on that and to avoid repetition the model underlying all three approaches is explained only once in the section on the multilevel model (except for approach-specific differences given in the paragraphs on each method). The supplementary R script implements all three methods using R and WinBUGS (Text S1).

#### Least squares ANOVA

Although the least squares ANOVA can be referred to as a mixed model, the only difference from an entirely fixed-effects ANOVA is in the assignment of the error terms for the F tests. However, as explained in the main text, one major limitation of mixed-model ANOVA is the difficultly involved in specifying these error terms. In particular, there are two alternative schools of thought that are usually explained with regard to a simple mixed model with one fixed factor, one random factor, the (random) interaction term and an overall residual error. Both approaches test the fixed effect against the interaction. The disagreement comes over testing the main effect of the random factor. The so-called constrained approach tests the main effect of a random factor against the overall residual while the unconstrained approach uses the interaction [23]. In biodiversity experiment the principle interest is usually in species richness (or a similar measure of diversity). This debate does not affect the inference for species richness effects since both approaches test the fixed effect against its interaction with the random factor (experiments). The interaction is tested against the overall residual (note that alternative tests against other interactions produce the same qualitative results for the data analysed here).

#### Mixed-Effects Model Analysis

One advantage of mixed-effects models is that there is greater agreement over how to test the random main effect: so far as we are aware, all MEM software produces results consistent with the unconstrained approach [24]. Our analysis has species richness as the only fixed-effect with all other explanatory variables and interactions (see below) treated as random effects. We used restricted maximum likelihood (REML) as opposed to standard maximum likelihood.

#### Bayesian Multilevel Model Analysis

We implemented the multilevel model analysis using the WinBUGS version of the BUGS family of statistical software based on similar earlier analyses [9], [53], [64]. BUGS stands for Bayesian inference Using Gibbs Sampling, a type of Markov chain Monte Carlo (MCMC) [28], [29], [30]. MCMC is sometimes simply described as a random walk in parameter space: parameter values are randomly varied and the likelihood estimated to build up a (posterior) distribution from which point estimates (the mean or median) and credible intervals (the appropriate quantiles) are derived.

The spatial blocking structure of our analysis consisted of plots nested within blocks and experimental sites. The two treatments, species richness and species composition, were applied at the plot level. When the same species (monoculture) or combination of species (polyculture) occurred at more than one site they were assigned the same code (that is, a mixtures of species A and B would be assigned the same numerical code regardless of experimental site). We also fitted the interactions between experimental site and species richness and between experimental site and species composition. Specifically, we modeled the response 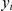 measured in plot 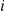 as follows:
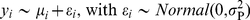
(1)


Hence, we assumed that the response in plot 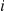 was normally distributed about a mean of 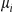 with plot-specific deviations 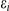. As usual, we assumed that the variability of these residuals 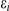 could be described by a zero-mean normal distribution with residual variance 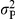. We assumed further that the plot-specific mean 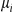 was made up of additive contributions (main effects) of experimental sites, block, species richness (log_2_ transformed) and species composition as well as of the interactions between experimental site and species richness and between experimental site and species composition:



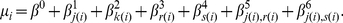
(2)


Here, the expected yield 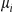 at plot 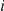 consisted of a grand mean 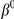 plus additive contributions 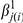 of experimental sites 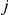 (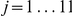), 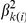 of block 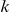 (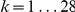), 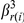 of species richness 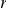 (continuous, log_2_ transformed), 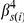 of species composition level (s = 1…294), 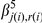 of the interaction between experimental sites 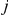 and species richness level 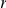, and 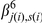 of the interaction between experimental sites 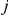 and the species composition level 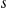.

To make sources of variation comparable, we followed Gelman (2005) and assumed that all six sets of effects were drawn from separate, independent, zero-mean normal distributions: 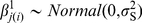
(3)

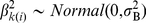
(4)

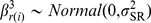
(5)

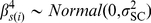
(6)

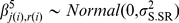
(7)

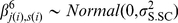
(8)


Hence, we estimated variance components for experimental sites (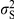), blocks within experimental sites (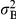), species richness (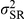), species composition (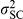) and for the interactions between experimental sites and species richness (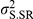) and experimental sites and species composition (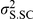), respectively.

As explained in the main text, Gelman [10] argues that for a description of the variance decomposition for all variables in a dataset, the finite-population variances 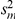 (the squared standard deviations of the factor level effects 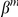 of a categorical explanatory variable 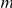) may be preferable to the super-population variances 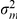. Hence, we based our inference about the magnitude of the effects of a factor on the finite-population standard deviation of the factor level effects, 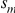, shown in Figure 3.

The model described in eq. 1–8 can be fitted using least squares, maximum likelihood or Bayesian statistical methods. In the supplementary material we present a script for implementing all three approaches using R and WinBUGS (Text S1). With the Bayesian approach inference in random-effects models is exact, whereas in frequentist analyses (e.g. with the R function lmer in the lme4 package) it is only approximate and understates the uncertainty in the estimates because of the use of only point estimates for the variance components [9]. We ran WinBUGS [28], [29] from R using the R2WinBUGS package [32] to fit our model using conventional Markov chain Monte Carlo (MCMC) simulation techniques. To introduce minimal prior information, we used conventional vague priors for all parameters (see supplementary R script), which typically yields values close to the maximum likelihood estimates [48].

We ran three independent Markov chains for 100,000 iterations, discarded the first 5,000 iterations as a burn-in period (i.e. when the outputs are still affected by the choice of the arbitrary starting points of the chains and hence unrepresentative of the target posterior distribution), and thinned the remainder by 1 in 200. From each Markov chain this yielded a sample of 475 random draws from the posterior distribution. Values of the Brooks-Gelman-Rubin statistic, 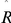, of less than 1.1 for all parameters suggested that the chains had reached convergence.

## Supporting Information

Text S1 (DOC)

Text S2 (TXT)
