# Supplementary material for: BUGS in the Analysis of Biodiversity Experiments: Species Richness and Composition Are of Similar Importance for Grassland Productivity
Source: PLoS One. 2011 Mar 2;6(3):e17434. doi: 10.1371/journal.pone.0017434 (PMC3047546; doi:10.1371/journal.pone.0017434)
Supplement: Text S1 — (DOC) [file pone.0017434.s001.doc]

**BUGS in the Analysis of Biodiversity Experiments:**

**Species Richness and Composition are of Similar Importance for Grassland Productivity**

**Andy Hector, Thomas Bell, Yann Hautier, Forest Isbell, Marc Kéry, Jasper** **van Ruijven, Peter Reich and Bernhard Schmid.**

**Text S1**

WinBUGS and R script used for this paper. For good references see: (Gelman and Hill 2007, Qian and Shen 2007)

**##### load libraries**

library(lattice)

library(ggplot2)

library(arm)

library(R2WinBUGS)

**##### load data**

MixEff <- read.table(file.choose(),header=T) # biodepthBioCONBioGenJenaWageningen.txt

str(MixEff)

**##### Making factors, transformations etc.**

levels(MixEff$Site) <- c("Biodepth Bayreuth", "Cedar Creek BioCON", "Biodepth Greece", "Biodepth Ireland", "Jena",

"Biodepth Portugal", "Biodepth Uk/Sheffield", "Biodepth UK/Silwood", "Biodepth Sweden", "Biodepth Switzerland", "BioGEN", "Wageningen")

MixEff$mix.crossed <- factor(MixEff$mix.crossed)

MixEff$SR.log2.p <- log(MixEff$sr,2)

MixEff$mass.p <- MixEff$mass

MixEff$Site <- relevel(MixEff$Site, ref=c("Biodepth Bayreuth", "Biodepth Greece", "Biodepth Ireland", "Biodepth Portugal",

"Biodepth Uk/Sheffield", "Biodepth UK/Silwood", "Biodepth Sweden", "Biodepth Switzerland", "Cedar Creek BioCON", "Jena", "BioGEN", "Wageningen"))

**#########################################################**

**# 1. Least squares mixed-model ANOVA**

**# Codes for the model**

ls1 <- lm(terms(mass.p ~ Site+Block+SR.log2.p+Site:SR.log2.p+mix.crossed+Site:mix.crossed, keep.order= TRUE), data= MixEff)

**# Codes to produce the Table 2**

anova(ls1) # then import in Excel and calculate the correct F and P values of the treatments with the correct error terms

**# Codes to produce the Figure 1**

p <- qplot(SR.log2.p, mass.p, geom= c("point", "smooth"), method = "lm",

data=MixEff, xlab=expression(paste("Species richness (log"["2"]," scale)")),ylab="Aboveground biomass (g)")

p + theme_bw() + scale_x_continuous(breaks=c(0,1,2,3,4,5), labels=c("1","2","4","8","16","32")) + facet_wrap(~ Site, ncol = 4)

**#########################################################**

**# 2. Mixed-effects model**

**# Codes for the model**

mem2 <- lmer(mass.p~ SR.log2.p +(1+SR.log2.p|Site)+(1|Block)+(1|mix.crossed)+(1|Site:mix.crossed), data= MixEff)

**# Codes to produce the Table 3**

# Fixed effects

anova(mem2)

# Random effects

model1 <- lmer(mass.p~ SR.log2.p +(1+SR.log2.p|Site)+(1|Block)+(1|mix.crossed)+(1|Site:mix.crossed), data= MixEff)

model2 <- lmer(mass.p~ SR.log2.p +(1+SR.log2.p|Site)+(1|Block)+(1|mix.crossed), data= MixEff)

model3 <- lmer(mass.p~ SR.log2.p +(1+SR.log2.p|Site)+(1|Block), data= MixEff)

model4 <- lmer(mass.p~ SR.log2.p +(1+SR.log2.p|Site), data= MixEff)

model5 <- lmer(mass.p~ SR.log2.p +(1|Site), data= MixEff)

anova(model1, model2, model3, model4, model5)

# get the log likelihood of the model without random terms from the least square ANOVA and calculate the associated Chi square and P values.

model6 <- lm(mass.p~ SR.log2.p, data= MixEff, na.action=na.omit)

logLik(model6)

# Chisq

Chisq <- 2*( logLik(model6)+ logLik(model5))

# P value from the Chisq distribution and the difference in degrees of freedom

pchisq(Chisq[1], 1)

**# Codes to produce the Figure 2**

# Create a data frame with the intercepts and slopes for each experimental site

Int <- coef(mem2)$Site[,1]

Slope <- coef(mem2)$Site[,2]

Site <- levels(MixEff$Site)

NewData <- data.frame(Int, Slope, Site)

NewData

# Get the average biomass for each species composition

MoltenMixEff <- melt(MixEff[1:7], id=c(1:5))

MixEff.mix <- cast(MoltenMixEff, Site+mix.crossed ~ variable, mean, na.rm=TRUE)

MixEff.mix <- MixEff.mix[!is.na(MixEff.mix$mass),]

MixEff.mix$SR.log2 <- log(MixEff.mix$sr,2)

head(MixEff.mix)

# Get the standard error of the mean for each species composition

Mix.coef <- coef(mem2)$`Site:mix.crossed`

a.se <- se.coef(mem2)$`Site:mix.crossed`[,1]

semLow <- MixEff.mix$mass - a.se

semUp <- MixEff.mix$mass + a.se

head(Mix.coef)

# Create a data frame with the intercepts and slopes for each experimental site

NewData.mix <- data.frame(MixEff.mix, a.se, semLow, semUp, Mix.coef)

names(NewData.mix) <- c("Site", "mix.crossed", "sr", "mass.p", "SR.log2.p", "a.se", "semLow", "semUp", "Intercept", "Slope")

head(NewData.mix)

# Create a data frame with the overall intercept and slope

fixef(mem2)

Intercept <- fixef(mem2)[1]

Slope <- fixef(mem2)[2]

NewData.fix <- data.frame(Intercept,Slope); NewData.fix

# Make the graph with qplot

p <- qplot(SR.log2.p,mass.p,facets=.~Site,xlab=expression(paste("Species richness (log"["2"]," scale)")),ylab="Mixture average aboveground biomass (g)",data= NewData.mix)

p + theme_bw() + geom_abline(data=NewData, aes(intercept=Int,slope=Slope)) + geom_linerange(aes(min=semLow, max=semUp)) +

geom_abline(data=NewData.fix, aes(intercept=Intercept,slope=Slope), colour="red") +

scale_x_continuous(breaks=c(0,1,2,3,4,5), labels=c("1","2","4","8","16","32")) + facet_wrap(~ Site, ncol = 4)

**#########################################################**

**# 3. WinBUGS**

**# Model**

## likelihood function

biodModel <- function () {

for (i in 1:n){

y[i] ~ dnorm (y.hat[i], tau.y)

y.err[i] <- y[i]-y.hat[i]

y.hat[i] <- int.SITESR[site[i]] + beta.SITESR[site[i]]*sr[i] + beta.SITE[site[i]] + beta.BL[block[i]] + beta.MIX[mix[i]] + beta.SITESC[mix[i], site[i]]

srM[i] <- int.SR + beta.SR*sr[i]

srI[i] <- gamma.SITESR[site[i]]*sr[i]

}

## Priors

int.SR ~ dnorm(0, 0.0001)

beta.SR <- mean(beta.SITESR[])

for (i.site in 1:n.site){

int.SITESR[i.site] ~ dnorm (0, 0.0001)

beta.SITE[i.site] ~ dnorm (0, tau.SITE)

beta.SITESR[i.site] ~ dnorm (0, tau.SITESR)}

for (i.bl in 1:n.bl){

beta.BL[i.bl] ~ dnorm (0, tau.BL)}

for (i.mix in 1:n.mix){

beta.MIX[i.mix] ~ dnorm (0, tau.MIX)

for (i.site in 1:n.site){

beta.SITESC[i.mix, i.site] ~ dnorm (0, tau.SITESC)}

}

sigma.BL ~ dunif(0,100)

sigma.MIX ~ dunif(0,100)

sigma.SITE ~ dunif(0,100)

sigma.SITESR ~ dunif(0,100)

sigma.SITESC ~ dunif(0,100)

sigma.y ~ dunif(0, 100)

tau.BL <- pow(sigma.BL, -2)

tau.MIX <- pow(sigma.MIX, -2)

tau.SITE <- pow(sigma.SITE, -2)

tau.SITESR <- pow(sigma.SITESR, -2)

tau.SITESC <- pow(sigma.SITESC, -2)

tau.y <- pow(sigma.y, -2)

## Calculating standard deviations of all variables

s.y <- sd(y.err[])

s.SR <- abs(beta.SR)*sd(sr[])

s.BL <- sd(beta.BL[])

s.MIX <- sd(beta.MIX[])

s.SITE <- sd(beta.SITE[])

s.SITESR <- sd(srI[])

s.SITESC <- sd(beta.SITESC[,])

## Derived quantities

gamma.0 <- int.SR + mean(sr[])

for (i.site in 1:n.site){

gamma.SITE[i.site] <- beta.SITE[i.site] - mean(beta.SITE[])

gamma.SITESR[i.site] <- beta.SITESR[i.site] - mean(beta.SITESR[])}

for (i.bl in 1:n.bl){

gamma.BL[i.bl] <- beta.BL[i.bl] - mean(beta.BL[])}

for (i.mix in 1:n.mix){

gamma.MIX[i.mix] <- beta.MIX[i.mix] - mean(beta.MIX[])

for (i.site in 1:n.site){

gamma.SITESC[i.mix, i.site] <- beta.SITESC[i.mix, i.site] - mean(beta.SITESC[,])}

}

# means

for (i.site in 1:n.site){

mean.SITE[i.site] <- mean(beta.SITE[])

mean.SITESR[i.site] <- mean(beta.SITESR[])}

for (i.bl in 1:n.bl){

mean.BL[i.bl] <- mean(beta.BL[])}

for (i.mix in 1:n.mix){

mean.MIX[i.mix] <- mean(beta.MIX[])

for (i.site in 1:n.site){

mean.SITESC[i.mix, i.site] <- mean(beta.SITESC[,])}

}

}

## Write the model: to run this program from R, we save the BUGS model in a text file

write.model(biodModel, file.path("C:/Program Files/R/R-2.8.1/library/R2WinBUGS/model/biodepth.txt"))

**# R function**

## Supply data for variables

bugs.in <- function(infile=MixEff){

y <- infile$mass

n <- length(y)

block <- as.numeric(ordered(infile$Block))

mix <- as.numeric(ordered(infile$Mix))

site <- as.numeric(ordered(infile$Site))

sr <- infile$SR.log2

n.bl <- max(block)

n.mix <- max(mix)

n.site <- max(site)

## Input data and initial values

## Initial values (for all unknown coefficients to be estimated)

## Three parallel MCMC chains to facilitate convergence diagnostic

bugs.dat <- list(n=n, n.bl=n.bl, n.mix=n.mix, n.site=n.site, y=y, sr=sr, block=block, mix=mix, site=site )

inits1 <- list(int.SR=1, beta.SITE = rep(0, n.site), beta.SITESR=rep(0, n.site),

beta.SR = 1, beta.BL = rep(0, n.bl), beta.MIX = rep(0, n.mix),

beta.SITESC=matrix(0, ncol=n.site, nrow=n.mix),

sigma.y =1, sigma.BL =1, sigma.MIX=1, sigma.SITE=1, sigma.SITESR=1, sigma.SITESC=1)

inits2 <- list(int.SR=0, beta.SITE = rep(1, n.site), beta.SITESR=rep(1, n.site),

beta.SR = 0, beta.BL = rep(1, n.bl), beta.MIX = rep(1, n.mix),

beta.SITESC=matrix(1, ncol=n.site, nrow=n.mix),

sigma.y =0, sigma.BL =0, sigma.MIX=1, sigma.SITE=0, sigma.SITESR=0, sigma.SITESC=0)

inits3 <- list(int.SR=0.5, beta.SITE = rep(0.5, n.site), beta.SITESR=rep(0.5, n.site),

beta.SR = 0.5, beta.BL = rep(0.5, n.bl), beta.MIX = rep(0.5, n.mix),

beta.SITESC=matrix(0.5, ncol=n.site, nrow=n.mix),

sigma.y =1, sigma.BL =0.5, sigma.MIX=0.5, sigma.SITE=0.5, sigma.SITESR=0.5, sigma.SITESC=0.5)

inits <- list (inits1, inits2, inits3)

parameters <- c("s.y","s.SR","s.BL","s.MIX","s.SITE","s.SITESR","s.SITESC",

"int.SITESR","int.SR",

"beta.SR", "beta.BL", "beta.MIX", "beta.SITESR", "beta.SITESC",

"gamma.0","gamma.BL","gamma.MIX","gamma.SITE","gamma.SITESR","gamma.SITESC",

"mean.SITE", "mean.SITESR", "mean.BL", "mean.MIX", "mean.SITESC")

return(list(para=parameters, data=bugs.dat, inits=inits))

}

## Create input and initial data

input.to.bugs <- bugs.in()

## Do the simulation

bugs.out.S <- bugs(input.to.bugs$data, input.to.bugs$inits,

input.to.bugs$para, file.path("C:/Program Files/R/R-2.8.1/library/R2WinBUGS/model/biodepth.txt"),

n.chains=3, n.iter=100000, n.thin=200, n.burnin=5000, DIC=F, debug=T)

**# Codes to produce the Table 4**

bugs.out.S$summary[1:7,]

# get 68% CRI using the quantile() function

res <- quantile(bugs.out.S$sims.list$s.y, prob = c(0.1587, 0.8413)) # residuals

sr <- quantile(bugs.out.S$sims.list$s.SR, prob = c(0.1587, 0.8413)) # species richness

bl <- quantile(bugs.out.S$sims.list$s.BL, prob = c(0.1587, 0.8413)) # block

mix <- quantile(bugs.out.S$sims.list$s.MIX, prob = c(0.1587, 0.8413)) # species composition

site <- quantile(bugs.out.S$sims.list$s.SITE, prob = c(0.1587, 0.8413)) # site

sitesr <- quantile(bugs.out.S$sims.list$s.SITESR, prob = c(0.1587, 0.8413)) # site x species richness interaction

sitesc <- quantile(bugs.out.S$sims.list$s.SITESC, prob = c(0.1587, 0.8413)) # site x species composition interaction

a16 <- as.numeric(c(res[1],sr[1],bl[1],mix[1],site[1],sitesr[1],sitesc[1]))

a84 <- as.numeric(c(res[2],sr[2],bl[2],mix[2],site[2],sitesr[2],sitesc[2]))

out.table <- cbind(bugs.out.S$summary[1:7,], a16, a84)

out.table

**# Codes to produce the Figure 3 (taken from Qiann & Shen 2007)**

summary.plot <- function(out.table, rows, ylab = NULL, xlab="values",

ymar=11, Mean=T){

Plot.data <- out.table[rows,]

plotting.region <- range(Plot.data[,c(3,7)])

par(mar=c(4,ymar,1,1))

plot(c(0, 200),

c(0.5,length(rows)+0.5), type="n",

xlab=xlab, ylab=" ", axes=F)

axis(1)

if (is.null(ylab))

axis(2, at=1:length(rows), labels=row.names(Plot.data), padj=0.5, las=1)

else axis(2, at=1:length(rows), labels=ylab, padj=0.5, las=1)

segments(x0=Plot.data[,3], x1=Plot.data[,7],

y0=1:length(rows), y1=1:length(rows), col="black")

segments(x0=Plot.data[,10], x1=Plot.data[,11],

y0=1:length(rows), y1=1:length(rows), lwd=3, col="black")

abline(v=0, col="grey")

if(Mean) points(Plot.data[,1], 1:length(rows), col='black', pch=19, cex=1, lwd=1)

else points(Plot.data[,5], 1:length(rows), col='black', pch=19, cex=1, lwd=1)

invisible()

mtext("Explanatory variable", side=3, adj=-0.41, cex=1, font=2)

mtext("Variance components", side=3, adj=0.5, cex=1, font=2)

}

summary.plot(out.table, c(1,7,4,6,2,3,5),

xlab="Standard Deviation",

ylab=c("Residuals", "SC x Experiment","Species composition (SC)", "SR x Experiment", "Species richness (SR)", "Block", "Experiment"))

**References**

Gelman, A., and J. Hill. 2007. Data Analysis Using Regression and Multilevel/Hierarchical Models, 2 edition. Cambridge Univ. Press, Cambridge.

Qian, S. S., and Z. Shen. 2007. Ecological applications of multilevel analysis of variance. Ecology **88**:2489-2495.
